# R-Flurbiprofen Reduces Neuropathic Pain in Rodents by Restoring Endogenous Cannabinoids

**DOI:** 10.1371/journal.pone.0010628

**Published:** 2010-05-13

**Authors:** Philipp Bishay, Helmut Schmidt, Claudiu Marian, Annett Häussler, Nina Wijnvoord, Simone Ziebell, Julia Metzner, Marco Koch, Thekla Myrczek, Ingo Bechmann, Rohini Kuner, Michael Costigan, Faramarz Dehghani, Gerd Geisslinger, Irmgard Tegeder

**Affiliations:** 1 pharmazentrum frankfurt/ZAFES, Clinical Pharmacology, Goethe-University, Frankfurt, Germany; 2 Department of Clinical Neuroanatomy, Institute of Anatomy, Goethe-University, Frankfurt, Germany; 3 Department of Experimental Neurobiology, Institute of Anatomy, Goethe-University, Frankfurt, Germany; 4 Department of Pharmacology, University of Heidelberg, Heidelberg, Germany; 5 Neuroplasticity Research Group, Department of Anaesthesia, Massachusetts General Hospital and Harvard Medical School, Boston, Massachusetts, United States of America; Dr. Margarete Fischer-Bosch Institute of Clinical Pharmacology, Germany

## Abstract

**Background:**

R-flurbiprofen, one of the enantiomers of flurbiprofen racemate, is inactive with respect to cyclooxygenase inhibition, but shows analgesic properties without relevant toxicity. Its mode of action is still unclear.

**Methodology/Principal Findings:**

We show that R-flurbiprofen reduces glutamate release in the dorsal horn of the spinal cord evoked by sciatic nerve injury and thereby alleviates pain in sciatic nerve injury models of neuropathic pain in rats and mice. This is mediated by restoring the balance of endocannabinoids (eCB), which is disturbed following peripheral nerve injury in the DRGs, spinal cord and forebrain. The imbalance results from transcriptional adaptations of fatty acid amide hydrolase (FAAH) and NAPE-phospholipase D, i.e. the major enzymes involved in anandamide metabolism and synthesis, respectively. R-flurbiprofen inhibits FAAH activity and normalizes NAPE-PLD expression. As a consequence, R-Flurbiprofen improves endogenous cannabinoid mediated effects, indicated by the reduction of glutamate release, increased activity of the anti-inflammatory transcription factor PPARγ and attenuation of microglia activation. Antinociceptive effects are lost by combined inhibition of CB1 and CB2 receptors and partially abolished in CB1 receptor deficient mice. R-flurbiprofen does however not cause changes of core body temperature which is a typical indicator of central effects of cannabinoid-1 receptor agonists.

**Conclusion:**

Our results suggest that R-flurbiprofen improves the endogenous mechanisms to regain stability after axonal injury and to fend off chronic neuropathic pain by modulating the endocannabinoid system and thus constitutes an attractive, novel therapeutic agent in the treatment of chronic, intractable pain.

## Introduction

Persistent intractable pain comes about as a sequel of peripheral or central nerve injury and is a major health problem. Conventionally used analgesics are often not sufficiently effective or their long-term usage is accompanied by side-effects, which seriously narrow the quality of life and lead to poor compliance and rejection of therapy. Various novel targets and compounds have been identified in recent years that might be useful in neuropathic pain syndromes [1], [2]. However, so far none of these has been approved for clinical use.

Here, we studied the R-enantiomer of a well-known non steroidal anti-inflammatory drug (NSAID), flurbiprofen-racemate [3], that has been used for decades as analgesic. Interestingly, R-flurbiprofen does not inhibit cyclooxygenase activity [4] and has been considered as the non-functional constituent of marketed flurbiprofen-racemate. However, R-flurbiprofen reduces inflammation [5] via inhibition of the transcription factor NF-κB [5] and is essentially free of the side effects typical to classical NSAIDs, such as gastrointestinal or renal toxicity [6]. Because of the anti-inflammatory efficacy and essential lack of toxicity R-flurbiprofen has been evaluated as a potential remedy in Alzheimer's disease with some success in clinical trials [7]. R-flurbiprofen also attenuates nociceptive behavior in rats [8] and pain in humans [9]. In these species, R-flurbiprofen is not inverted to its cyclooxygenase inhibiting S-enantiomer. Previously, it has been suggested based on in vitro experiments that some NSAIDs modify endocannabinoid breakdown [10]. It is unknown whether such effects occur in vivo. However, it has been shown that inhibition of endocannabinoid metabolism via specific inhibition of fatty acid amide hydrolase (FAAH) or monoacylglycerol lipase (MAGL) reduces pain in inflammatory and neuropathic pain models [11], [12], without producing diverse side effects, such as temporary memory impairment, addiction and psychotropic effects, which are associated with agonists at cannabinoid-1 (CB1) receptors in forebrain circuits.

Since fortification of endogenous pain defense has emerged as a valuable strategy particularly in chronic pain we probed antinociceptive mechanisms of R-flurbiprofen in models of peripheral nerve injury and found that R-flurbiprofen reduces neuropathic pain in rodents by normalizing pathologically reduced endocannabinoid levels in DRGs, spinal cord and frontal cortex without direct CB1-mediated central effects. As a consequence, R-flurbiprofen reduces glutamate release in the dorsal horn evoked by nerve injury and prevents the development of the neuro-aggressive microglia phenotype. The endogenous defense against pain is thus potentiated, without tolerance or psychotropic side effects. As R-flurbiprofen is already known to be safe and neuroprotective in humans it may be used as “add-on” treatment for chronic neuropathic pain.

## Methods

### Animals and treatments

All experiments adhered to the guidelines of the Committee for Research and Ethical Issues of the International Association for the Study of Pain (IASP), were approved by the local Ethics Committee for Animal Research (Darmstadt, Germany) and adhered to the guidelines of GV-SOLAS for animal welfare in science (permission numbers F95/22, F143/26).

Male Sprague Dawley rats (Charles River, Sulzfeld, Germany) weighing 150–200 g or 8–12 week old C57Bl6 mice were used in most experiments. Mice deficient of the cannabinoid CB1 receptor specifically in a primary sensory neurons (SNS-CB1^−/−^) were generated via cre-loxP-mediated recombination by mating mice carrying the CB1-flox allele (CB1^fl/fl^) with mice expressing cre recombinase under control of the Nav1.8 promoter (SNS-cre). The SNS-cre mice enable gene recombination commencing at birth selectively in Nav1.8-expressing sensory neurons, without affecting gene expression in the spinal cord, brain or any other organs in the body [13]. Genotyping was done on mouse genomic tail DNA as described [13] using primers: for sense strand 5′-GCTGTCTCTGGTCCTCTTCTTAAA-3′ and for anti-sense 5′-GGTGTCACCTCTGAAAACAGA-3′ to detect CB1 flox allele and for sense strand 5′-GAAAGCAGCCATGTCCAATTTACTGACCGTAC-3′ and for anti-sense strand 5′-GCGCGCCTGAAGATATAGAAGA-3′ to detect SNS-cre transgene expression. Littermates were used in all experiments to control for background effects.

The enantiomers of flurbiprofen (2-(2-fluoro-4-biphenylyl)-propionic acid) were supplied by PAZ Arzneimittelentwicklungsgesellschaft (Frankfurt/Main, Germany). The optical purity of both enantiomers was >99%. Flurbiprofen enantiomers were dissolved in phosphate buffer and 2.5, 4.5 or 9 mg/kg were injected twice daily intraperitoneally or 10 mg/kg/d was administered perorally in the drinking water. The latter was used in experiments which assessed the inhibitory effects of the CB1 and CB2 cannabinoid receptor antagonists, AM251 and AM630 (Cayman Chemicals). The cannabinoid antagonists were dissolved in 1∶1 DMSO/sodium chloride and injected intraperitoneally (3 mg/kg). Gabapentin (1-(aminomethyl)-cyclohexane acetic acid) was obtained from Sigma-Aldrich and used at 25 mg/kg per day. Control animals received the respective vehicles.

### Nerve injury models

Surgery was carried out under 1.5–2% isoflurane anesthesia. For spared nerve injury (SNI), two of the three peripheral branches of the sciatic nerve, the common peroneal and the tibial nerves, were ligated with silk (6–0) and distally transected, leaving the sural nerve intact [14]. For chronic constriction injury (CCI), we tied three silk ligatures (6–0) around the proximal sciatic nerve constricting the nerve by about 30–50% [15].

### Behavioral experiments

All tests were performed by an investigator blinded for the treatment or genotype of the animals. After habituation, we determined the latency for paw withdrawal to von Frey-like filament using a Dynamic Plantar Aesthesiometer (Ugo Basile, Italy) to assess mechanical allodynia. The steel rod was pushed against the paw with linear ascending force (0–50 grams for rats and 0–5 grams for mice over 10 seconds) until a strong and immediate withdrawal occurred. The paw withdrawal latency was the mean of three consecutive trials with intervals of at least 30 sec. To measure cold allodynia, we recorded the latency for paw withdrawal, paw licking or jumping after placing rats or mice onto a Cold Plate held at 5°C (AHP-1200CPHC, Teca, USA) and by employing the acetone test. The time rats or mice spent licking, lifting or shaking the nerve injured paw after application of a drop of acetone to the plantar side were recorded for a period of 90 seconds starting right after acetone application. Heat hyperalgesia was assessed by recording temperature withdrawal thresholds on a Hot Plate which allows for temperature gradient analyses. The starting temperature was set at 28°C and increased by 10°C per minute up to paw withdrawal. The cut off temperature was 54°C.

### Core temperature

Mouse telemetry was used to analyze the time courses of mouse body temperature. Two days before analysis a radio telemetry transmitter (Emitter 4000 system, Mini Mitter, Bend, OR) was inserted into the intraperitoneal cavity for continuous recording of the core body temperature under isoflurane anesthesia. For measurements each cage was placed on a receiver and radio telemetry signals emitted by the implanted transmitter were continuously monitored in intervals of 5 min using the Vital view software (Mini Mitter). After 60-min baseline recordings drugs or vehicle were injected and the temperature recorded for further 3.5 hours.

### Spinal cord microdialysis

Microdialysis was employed to assess glutamate release in the dorsal horn of the spinal cord. A dialysis probe was constructed from a polyacrylonitrile hollow fiber (AN69, OD 200 µm; molecular mass cutoff ∼40 kDa; Hospal, Germany), a polyethylene (PE) inlet tube (ID 0.26 µm, OD 0.6 µm) and a micro pin for insertion. We inserted the catheter during 1.5–2% isoflurane anesthesia through vertebra Th13 after drilling a small hole, which approximates L4-L5 in the rat lumbosacral spinal cord, as described [16]. The catheter was connected to a CMA 100 microdialysis pump and perfused with artificial cerebrospinal fluid (ACSF, composed of (in mM): 141.7 Na^+^, 2.6 K^+^, 0.9 Mg^2+^, 1.3 Ca^2+^, 122.7 Cl^−^, 21 HCO_3_^−^, 2.5 HPO_4_^2−^, and 3.5 dextrose, aerated with 5% CO_2_ in 95% O_2_ to adjust pH to 7.2) at a flow rate of 1 µl/min. During a 90 min dialysis equilibration period anesthesia was switched to urethane (1.2 g/kg i.p.). At the end of baseline sampling of dialysates (12×5 min intervals) we exposed the mid sciatic nerve and transected it for axotomy or applied 1% capsaicin cream manufactured by the local pharmacy directly onto the nerve. Small sponges prevented leakage into surrounding tissue. The wound was loosely closed to prevent desiccation and dialysates sampled for 14×5 min intervals. At completion, the correct placement of the microdialysis catheter (dorsal horn, L4/5) was confirmed by dye injection and microscopic inspection.

We analyzed glutamate dialysate concentrations with a CMA 600 Analyzer (CMA, Stockholm, Sweden) according to the manufacturer's instructions. The device is used for routine clinical analysis of dialysate glutamate concentrations employing a glutamate oxidase/peroxidase two-step enzymatic assay and subsequent photometric quantification of the end-product quinonediimine at 546 nm (lower detection limit 1 µM).

### Microarray hybridization and quantitative RT-PCR

Total RNA was extracted from homogenized tissue according to the protocol provided in the RNAeasy tissue Mini Kit (Qiagen, Hilden, Germany), reverse transcribed using poly-dT as a primer to obtain cDNA fragments. cDNA fragments were amplified by PCR and cloned into the pCR4 vector (TA cloning Kit, Invitrogen). Biotinylated cRNA was produced by in vitro transcription and used for hybridization on the Affymetrix RGU34A chip as described [17], [18]. The data are MIAM compliant.

Quantitative real-time PCR was performed using the Sybrgreen detection system with primer sets designed on Primer Express. Specific PCR product amplification was confirmed with gel electrophoresis. Transcript regulation was determined using the relative standard curve method per manufacturer's instructions (Applied Biosystems). For each time point 3 samples of pooled tissue of 3 animals was analyzed.

### Western blot analysis and enzyme immune assays

Tissue samples were homogenized in Phosphosafe® (Novagen) cell lysis and extraction buffer, centrifuged at 16000 rpm for 5 min and the supernatant transferred to a new tube. For cannabinoid receptor extraction the Compartmental Protein Extraction Kit (Millipore) was used. Proteins were separated by SDS-PAGE (30 µg/lane) and transferred onto nitrocellulose membranes (Amersham Pharmacia Biotech) by wet-blotting. The blots were incubated with primary antibodies directed against CB1 (1∶500, Cayman Europe), CB2 (1∶500 Cayman Europe), FAAH (1∶1000, SantaCruz Biotechnology), NAPE-PLD (1∶500, Novus), Iba-1 (1∶500; Wako, Neuss, Germany), phospho-p38 and total p38 (Cell Signaling), CD68 (Abcam), beta-actin (SantaCruz Biotechnology), Hsp90 (SantaCruz Biotechnology) or extracellular signal-regulated kinase 2 (ERK-2; 1∶10000; SantaCruz Biotechnology) according to standard procedures. ERK-2, HSP-90 or beta-actin served as loading controls. After incubation with secondary antibodies conjugated with IRDye 680 or 800 (1∶10000; LI-COR Biosciences, Bad Homburg, Germany), blots were visualized and analyzed on the Odyssey Infrared Imaging System (LI-COR Biosciences). Triplicate experiments and duplicate blots each were obtained. The ratio of the respective protein band to the loading control was used for semi-quantitative analysis. Signal intensity of phospho-p38-immunoreactive bands was normalized relative to total p38. To further quantify p38 phosphorylation by EIA, we used a TiterZyme Enzyme Immunometric Assay Kit (AssayDesigns) following instructions provided by the manufacturer.

For analysis of PPARγ activity we employed a transcription factor assay kit (Actif Motif) that allows for the detection and quantification of transcription factor activation by a combination of specific oligonucleotide binding and subsequent detection with a specific antibody. Nuclear extracts were prepared from DRGs, spinal cord and forebrain cortex using a Nuclear-Cytosol Fractionation Kit (PromoKine) and were then analyzed with the PPARγ-kit as recommended by the manufacturer.

### Endocannabinoid analysis

Tissue pieces of 1–2 mg were weighed, homogenized and extracted by liquid-liquid extraction and the reconstituted samples were analyzed for arachidonoyl ethanolamide (anandamide, AEA), palmitoyl ethanolamide (PEA), 2-arachidonoylglycerol (2-AG), 1-arachidonoylglycerol (1-AG) and oleoyl ethanolamide (OEA). The respective deuterated substances AEA-d8, PEA-d4, 2-AG-d5, 1-AG-d5 and OEA-d2 were used as internal standards. We performed 2 cycles of ethylacetate extraction. 800 µl of ethylacetate was added to the homogenate, vortexed and centrifuged for 5 min at 10,000 g. The organic phase was removed and the extraction repeated. Ethylacetate fractions were combined and evaporated at a temperature of 45°C under a gentle stream of nitrogen. The residues were reconstituted with 100 µl of methanol, frozen at −20 degrees for 12 hours, and centrifuged for 5 min at 10,000 g. The residues were transferred in glass vials and 20 µl were injected into the LC-MS/MS system.

HPLC analysis was done under gradient conditions using a Luna HST C18 column (100 mm L×2 mm ID, 2.5 µm particle size, Phenomenex, Aschaffenburg, Germany). MS and MS/MS analyses were performed on an API 5000 triple quadrupole mass spectrometer with a Turbo V source (Applied Biosystems, Darmstadt, Germany) in the negative ion mode. Precursor-to-product ion transitions of m/z 346→259 for AEA, m/z 354→86 for AEA-d8, m/z 298→268 for PEA, m/z 302→272 for PEA-d4, m/z 377→303 for 2-AG and 1-AG, m/z 382→303 for 2-AG-d5 and 1-AG-d5, m/z 324→86 for OEA, and m/z 326→86 for OEA-d2 were used for the multiple reaction monitoring (MRM) with a dwell time of 75 ms. Concentrations of the calibration standards, quality controls and unknowns were evaluated by Analyst software (version 1.4; Applied Biosystems, Darmstadt, Germany). Variations in accuracy and intra-day and inter-day precision (n = 6 for each concentration, respectively) were <15% over the range of calibration.

### Immunofluorescence

Terminally anesthetized mice were transcardially perfused with 0.9% saline followed by 4% paraformaldehyde (PFA) in 0.1 M phosphate buffered saline (PBS, pH 7.4). The L4 and L5 spinal cord segments, L4 and L5 DRGs and sciatic nerves were dissected, postfixed for 2 h in PFA and transferred into sucrose (20% in PBS) for overnight cryoprotection at 4°C. We embedded the tissue in Tissue-Tek® O.C.T. Compound (Science Services, Munich, Germany) and cut 14 µm transverse sections of DRGs on a cryotome and mounted them on glass slides. Spinal cord tissue was sectioned at 30 µm on a vibratome and free floating sections were used for immunostainings. Sections were permeabilized for 5 min in PBST (0.1% Triton X-100 in PBS), blocked for 1 h with 10% normal goat serum and 3% bovine serum albumin (BSA) in PBST or with 1% blocking reagent (Roche Diagnostics) in PBST, and incubated overnight at 4°C with primary antibodies (IBA-1 DAKO, GFAP and NeuN Chemicon, CD11b Serotec, CD68 Abcam, CB1 Frontier Sciences) dissolved in 3% BSA or 1% blocking reagent in PBST. After washing in PBS, sections were incubated for 2 h at room temperature with species-specific secondary antibodies conjugated with Alexa Fluor 488 or 350 (Invitrogen, Karlsruhe, Germany) or Cy3 (Sigma-Aldrich, Munich, Germany). After immunostaining, slides were immersed for 5 min in 0.06% sudan black B (in 70% ethanol) to reduce lipofuscin-like autofluorescence [19], rinsed in PBS and cover slipped in Flouromount G (Southern Biotech, Birmingham, AL). Images were obtained using an Eclipse E600 microscope (Nikon, Düsseldorf, Germany) equipped with a Kappa DX 20 H camera and Kappa Image Base software (Kappa, Germany). Confocal images were obtained with a Zeiss LSM 510 laser scanning microscope (Zeiss, Germany).

### Image analysis

We used ImageJ software (version 1.39, National Institutes of Health) for quantification of CD11b immunoreactivity. The observer was blinded for the treatment. The mean pixel values per area were determined from 5 not overlapping areas in the dorsal horn using the polygon tool and background-corrected. Results are expressed as mean pixel intensity of each group and statistically compared by Student's t-test, *P*<0.05.

### Statistics

We used SPSS 17.0 for statistical evaluation. Data are presented as means ± SEM (behavior data) or SD (biochemical data). To compare the neuropathic pain behavior between groups we calculated the area under the nociception versus time curve (AUC) and subjected AUCs to one-way ANOVA of Student's t-tests. Immunostaining and western blot results were analyzed with Student's t-tests. *P* was set at 0.05 for all statistical comparisons.

## Results

### R-flurbiprofen reduces neuropathic pain-like behavior

**Figure 1:**
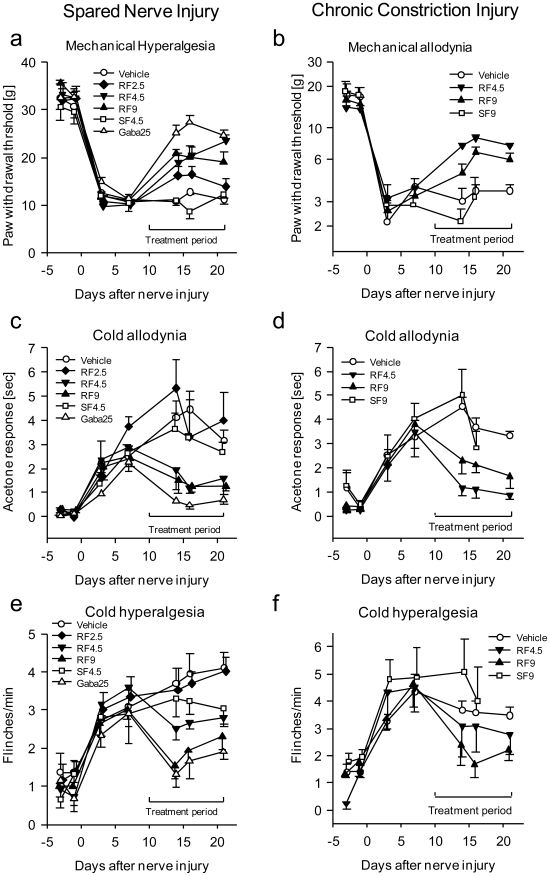
Time course of the nociceptive behavior in the Spared Nerve Injury (SNI, left panel) and the Chronic Constriction Injury (CCI, right panel) model of neuropathic pain. Rats were treated with R-flurbiprofen, S-flurbiprofen, gabapentin or vehicle (n = 6 per group). The daily treatment (twice daily i.p.) started 10 days after nerve injury. **a** Mechanical hyperalgesia was assessed by recording the paw withdrawal threshold to stimulation with a Dynamic Plantar Aesthesiometer, **b** mechanical allodynia as paw withdrawal threshold to von Frey hairs, **c, d **cold allodynia as response time in the acetone test and **e, f** cold hyperalgesia by counting withdrawal reactions during exposure to a cold plate at 2°C. Comparison of the areas under the effect x time curves revealed statistically significant differences between R-flurbiprofen and gabapentin treated animals compared with vehicle or S-flurbiprofen. For R-flurbiprofen 4.5 or 9 mg/kg twice daily provided statistically significant antinociceptive effects. For SNI, respective *P* values were 0.002 for mechanical, 0.026 for cold allodynia, 0.018 for cold hyperalgesia. For CCI, *P* values were 0.001 mechanical, 0.041 cold allodynia, n.s. for cold hyperalgesia.

**Figure 2:**
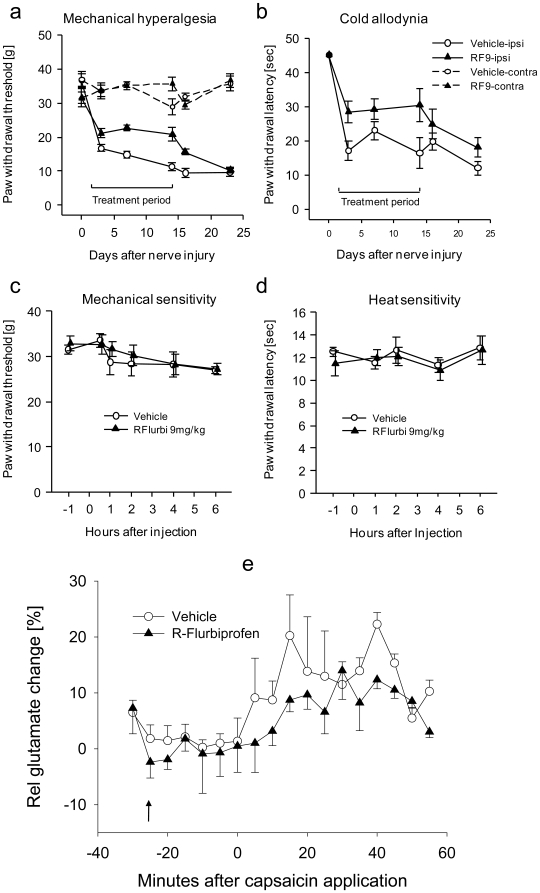
Time course of the nociceptive behavior in nerve-injured and naïve rats treated with R-flurbiprofen or vehicle (n = 6 per group) and glutamate release in the dorsal horn of the spinal cord. **a** In SNI, the daily drug treatment started the day after nerve injury. Mechanical hyperalgesia was assessed by recording the paw withdrawal threshold to stimulation with a Dynamic Plantar Aesthesiometer, **b** cold allodynia as paw withdrawal latency after exposure to a 10°C cold plate. Comparison of the areas under the effect x time curves revealed statistically significant differences between R-flurbiprofen and vehicle treatment (*P*<0.05). **c** In naïve rats mechanical sensitivity was assessed with a Dynamic Plantar Aesthesiometer, and **d** heat sensitivity as withdrawal latency to noxious heat stimulation in the Hargreaves test. R-flurbiprofen was injected at time zero, it had no effect. **e** Time course of glutamate release in the dorsal horn of the lumbar spinal cord in rats treated with 9 mg/kg R-flurbiprofen i.p. or vehicle 30 min before application of capsaicin cream (0.1%) onto the exposed sciatic nerve (time “zero”). Glutamate was analyzed in microdialysates from the dorsal horn in a colorimetric enzyme assay. The peak glutamate release after capsaicin application differed significantly between the treatment groups (n = 6; *P*<0.05).

We analyzed the effects of R-flurbiprofen in two rat models of neuropathic pain, the Spared Nerve Injury (SNI) and the Chronic Constriction Injury (CCI) model over two separate application regimes - first, at the onset of neuropathy and second, after neuropathic pain reached peak levels. In both models, neuropathy evokes a marked hypersensitivity to heat, cold and pressure, resulting in pain in response to innocuous stimuli (allodynia). Compared to vehicle or S-flurbiprofen, R-flurbiprofen-treated animals demonstrated a significant reversal of nociceptive thresholds towards normal pre-injury levels in the SNI (Fig. 1 a, c, e) as well as the CCI model (Fig. 1 b, d, f), showing that nociceptive hypersensitivity towards mechanical and cold stimulation was significantly ameliorated. The antinociceptive efficacy of R-flurbiprofen was dose-dependent and comparable with effects of gabapentin, which is one of the most effective drugs in the therapy of neuropathic pain. We observed therapeutic effects at daily doses of 2×4.5 and 2×9 mg/kg R-flurbiprofen (*P*<0.05). In both models, antinociceptive efficacy was achieved after full development of allodynia (Fig. 1). Allodynia was also prevented if treatment started directly after the nerve lesion (Fig. 2 a, b; *P*<0.001 mechanical; *P* = 0.029 cold allodynia). R-flurbiprofen did not change the sensitivity to mechanical or thermal stimulation in naïve animals, indicating lack of effects on normal sensory functions (Fig. 2 c, d). As expected, the S-enantiomer, S-flurbiprofen, produced gastrointestinal toxicity due to cyclooxygenase inhibition so that the treatment had to be stopped after seven days in several animals. In contrast, as shown previously [8], gastrointestinal toxicity did not occur with R-flurbiprofen as assessed by macroscopic inspection of the gastric mucosa at the end of the treatment period (data not shown).

### R-flurbiprofen reduces nerve injury evoked glutamate release in the spinal cord

Excessive and sustained glutamate release in the spinal cord contributes to the development of neuropathic pain [20], [21] and is inhibited by activation of presynaptic cannabinoid CB1 receptors [22]. We therefore analyzed by *in vivo* microdialysis whether R-flurbiprofen modifies stimulated glutamate release in the dorsal horn of the spinal cord. Baseline glutamate levels were similar in vehicle and R-flurbiprofen treated rats. Application of capsaicin, a nociceptive irritant and agonist at TRPV1, onto the sciatic nerve evoked an immediate glutamate raise, that was maintained for at least one hour and characterized by a double-peak (Fig. 2 e), as shown previously for formalin-evoked glutamate release [23]. R-flurbiprofen treated rats demonstrated a significant reduction in capsaicin-evoked glutamate release (Mann Whitney U comparison of peak release, *P* = 0.05; Fig. 2 e). We used capsaicin stimulation instead of axotomy in these experiments because capsaicin provides a longer lasting, more robust raise of glutamate [20].

### Effects of R-flurbiprofen on nerve injury evoked alterations of endocannabinoid synthesis and metabolism

**Figure 3:**
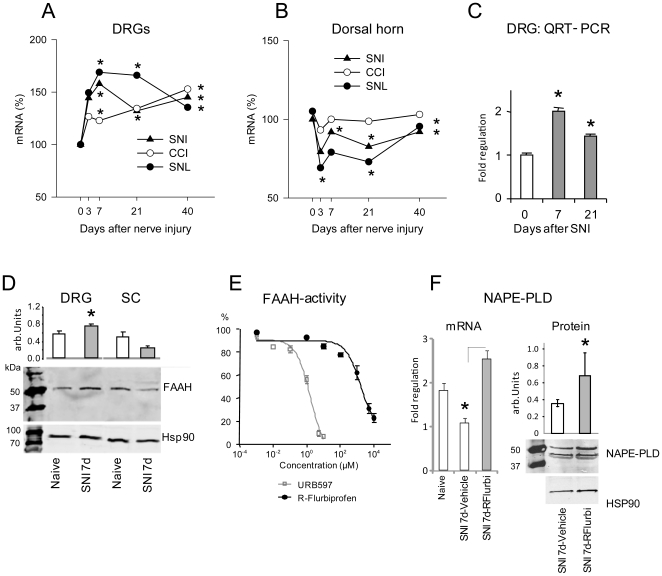
Expression and activity of fatty acid amide hydrolase (FAAH) and NAPE-PLD in DRGs and spinal cord after nerve injury. **a**, **b** Time course of FAAH mRNA levels in the L5 DRGs and the dorsal horn of the lumbar spinal cord ipsi lateral to a sciatic nerve lesion in three different models of neuropathic pain, i.e. the spared nerve injury, SNI, the chronic constriction injury, CCI and the spinal nerve ligation, SNL analyzed by Affymetrix U34 microarray in triplicate. Pooled samples of three animals each were used. The asterisks indicate statistically significant results with *P*<0.05. **c** Results were confirmed in the SNI model in mice by quantitative RT-PCR and **d** protein analysis in Western Blots. **e** Concentration dependent in vitro inhibition of recombinant FAAH by R-flurbiprofen and the FAAH specific inhibitor URB597 **f** QRT-PCR and Western Blot analysis of NAPE-PLD mRNA and protein expression in DRGs 7 days after SNI and treatment with R-flurbiprofen 4.5 mg/kg twice daily or vehicle. For QRT-PCR and Western Blot analysis pooled samples of each 3 mice were used and two independent experiments were performed. Mice were treated with 4.5 mg/kg R-flurbiprofen or vehicle twice daily (representative images; n = 6 per group).

The fast and sustained relief of behavioral correlates of neuropathic pain directly following R-flurbiprofen administration in vivo and the suppression of capsaicin-evoked glutamate release by R-flurbiprofen suggested a mechanism with rapid onset and creating a lasting impact on the underlying pathophysiology, both in line with the hypothesis of cannabinoid mediated effects. In a microarray screen of gene regulation in the DRGs and spinal cord, we found a long lasting upregulation of FAAH expression in the DRGs in three models of sciatic nerve injury (Fig. 3 a) associated with a drop of endocannabinoids. In the dorsal horn of the spinal cord, FAAH mRNA levels were slightly reduced at 3 days and subsequently returned to baseline levels (Fig. 3 b). We confirmed the time course of FAAH regulation in DRGs and spinal cord after SNI at the level of mRNA via quantitative RT-PCR (Fig. 3 c) and protein via Western Blot analysis (Fig. 3 d). At high concentrations R-flurbiprofen inhibited the activity of recombinant, purified FAAH; the IC50 of R-flurbiprofen was 1580 µM (CI 95%: 809.0 to 3096) as compared to an IC_50_ of 1.71 µM for the specific FAAH inhibitor, URB597 (CI 95%: 1.150 to 2.552) (Fig. 3 e). Although, *in vitro* data with recombinant enzyme cannot be directly translated into *in vivo* efficacy, it appears unlikely that direct inhibition of FAAH enzymatic activity is the only mechanism by which R-flurbiprofen attenuates neuropathic pain.

**Figure 4:**
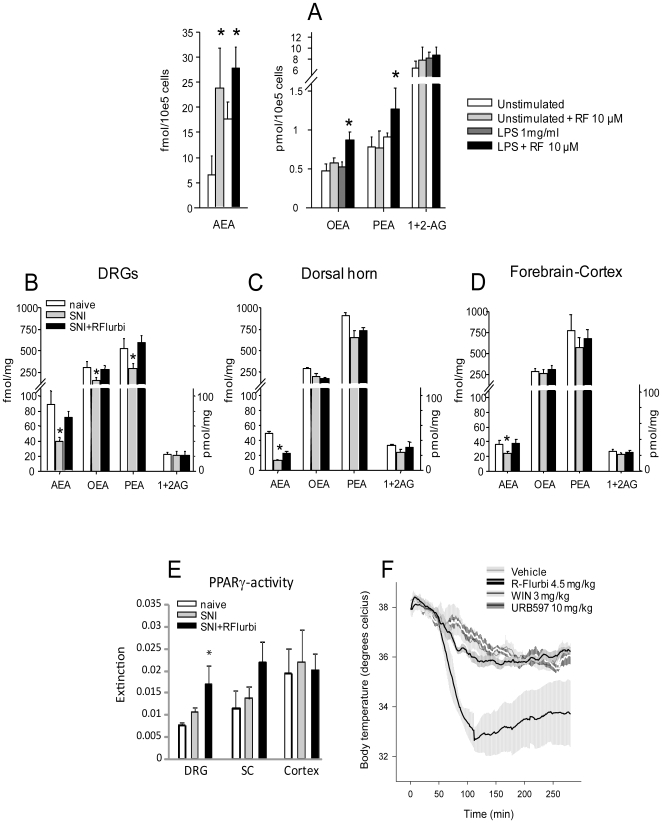
Modulation of endocannabinoid levels by R-flrubiprofen in microglial cells (EOC20) and tissue. **a** Endocannabinoids in EOC20 at baseline and after stimulation with LPS (1 mg/ml) with or without treatment with R-flurbiprofen (10 µM); n = 6 experiments per group. **b** Endocannabinoids in ipsi lateral L4/5 DRGs, **c** ipsi lateral dorsal horn of the spinal cord **d** and contra lateral forebrain cortex in the SNI model 7 days after nerve injury and treatment with 4.5 mg/kg R-flurbiprofen or vehicle twice daily in mice (n = 8 per group; *P*<0.05). AEA, anandamide; OEA, oleylethanolamine; PEA, palmitoylethanolamine; 1AG and 2AG, 1- and 2-arachidonoylglycerol were analyzed by LC-MS/MS. *P*<0.05 for all tests. **e** PPARγ activity in nuclear protein extracts of L4/5 DRGs, spinal cord and forebrain cortex in naïve mice and in nerve injured mice 7 days after SNI. Mice were treated with vehicle or 4.5 mg/kg R-flurbiprofen twice daily. Results represent pooled extracts of 6 mice in each group. **f** Core body temperature in mice (mean ± s.e.m. shadowed area) treated with the CB1 agonist, WIN55,212-2, the FAAH inhibitor URB597, R-flurbiprofen and respective vehicles (n = 6 per group). Vehicles 10% or 50% DMSO did not produce changes of the core temperature as compared to phosphate buffered saline and were summarized. The decrease of core temperature in vehicle treated mice is caused by resting during the day. Hypothermia in mice is defined as core temperature <35°C.

Despite weak FAAH inhibition, R-flurbiprofen significantly increased anandamide levels in unstimulated and LPS-stimulated microglia *in vitro* (Fig. 4 a). The levels of palmitoylethanolamine (PEA) and oleylethanolamine (OEA) that are also metabolized by FAAH were only increased in LPS-stimulated cells. Owing to these intriguing observations, we analyzed N-arachidonoyl-phosphatidylethanolamine phospholipase D (NAPE-PLD), the enzyme involved in anandamide synthesis. Anandamide is generated through phosphodiesterase-mediated cleavage of a cell membrane phospholipid precursor, N-arachidonoyl-phosphatidylethanolamine (NAPE) which is generated by the enzymatic transfer of arachidonic acid to the amine group of phosphatidyl-ethanolamine. NAPE cleavage to anandamide is catalyzed by NAPE-PLD [24]. RT-PCR and Western Blots revealed a decrease in NAPE-PLD expression in DRG neurons after sciatic nerve injury (Figures 3 f). This downregulation was completely prevented in R-flurbiprofen-treated animals (Figures 3 f).

**Figure 5:**
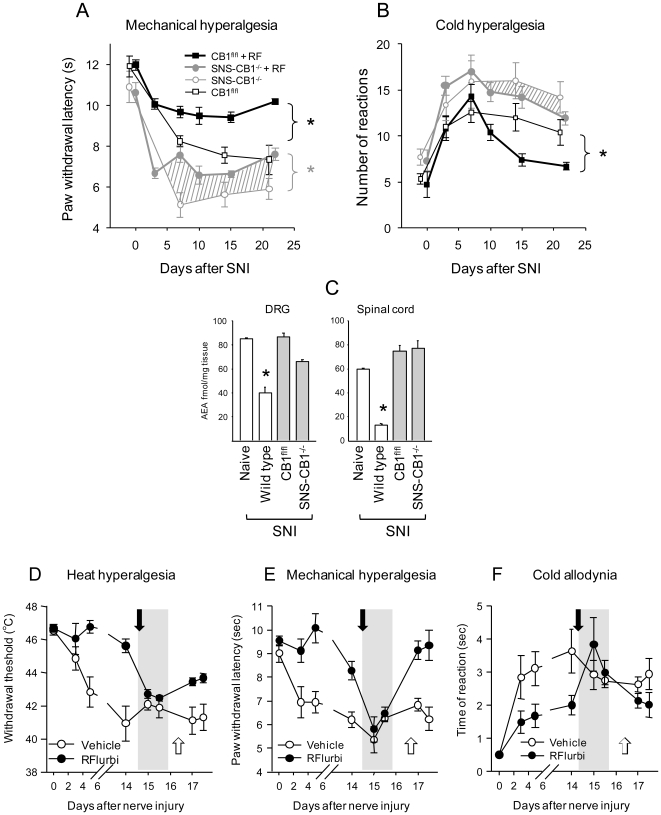
Time course of the nociceptive behavior in the SNI model of neuropathic pain in mice deficient of the CB1 receptor in DRG neurons (SNS-CB1^−/−^) compared with control mice carrying the floxed CB1 alleles (CB1^fl/fl^). Mice were treated with R-flurbiprofen (4.5 mg/kg twice daily) or vehicle (n = 8 per group). The daily drug treatment started the day after nerve injury. **a** Mechanical hyperalgesia was assessed by recording the paw withdrawal latency to stimulation with a Dynamic Plantar Aesthesiometer, **b** cold hyperalgesia as counts of paw withdrawal reactions after exposure to a 4°C cold plate. Comparison of the time courses by ANOVA revealed statistically significant differences between R-flurbiprofen and vehicle treatment in CB1^fl/f^ mice in both tests. In SNS-CB1^−/−^ mice R-flurbiprofen significantly reduced mechanical hyperalgesia but not cold hyperalgesia (n = 8 per group, P<0.05). The grey hatched areas indicate the difference in R-flurbiprofen and vehicle treated SNS-CB1^−/−^ mice. **c** Anandamide (AEA) levels in L4/5 DRGs and dorsal horn of the spinal cord in SNS-CB1^−/−^ and CB1^fl/fl^ mice treated with R-flurbiprofen or vehicle (n = 8, * indicates *P*<0.05). **5d, e, f** Effects of CB1 and CB2 cannabinoid receptor antagonists, AM251 and AM630, respectively on R-flurbiprofen mediated antinociception after nerve injury in the SNI model. R-flurbiprofen or vehicle was continuously administered in the drinking water starting after SNI surgery up to the end of the observation period. Fifteen days after SNI AM251 (3 mg/kg) and AM630 (3 mg/kg) were injected i.p. in R-flurbiprofen treated mice (black arrow) and heat (d), mechanical (e) and cold (f) pain analyzed 1 and 3 hours after AM251 and AM630 injection (grey area). To assess effects of AM251 and AM630 alone the drugs were injected (each 3 mg/kg) in vehicle treated mice at day 17 (white arrow) and nociception analyzed 1 and 3 hours after drug injection.

As a result of the adaptations of cannabinoid synthesizing and metabolizing enzymes after nerve injury, we observed that levels of anandamide, OEA and PEA in the DRGs were considerably reduced by about 50% after sciatic nerve injury (ANOVA *P*<0.001 for AEA, *P* = 0.045 for OEA and *P* = 0.027 for PEA). We analyzed the endocannabinoids in the DRG and not at the site of nerve damage because the latter is confounded by the endocannabinoid levels of infiltrating leukocytes in the periphery and dominated by 2-AG [13]. The observed drop of endocannabinoid levels in the DRGs was evident at 7 days after nerve injury (Fig. 4 b). It was maintained for at least 5 weeks (Fig. 5 c), i.e. lasting longer than the down-regulation of cannabinoid-1 receptor in the DRGs, which had a maximum at 3–7 days and then gradually returned to normal levels (Suppl. Fig. S1 a, S1 b). In the spinal cord dorsal horn and forebrain, anandamide levels showed a similar decrease as in the DRGs (Fig. 4 c, d) despite the observed drop of FAAH expression (Fig. 3). OEA, PEA and 2-AG were not significantly reduced. At both sites, R-flurbiprofen treatment restored anandamide levels, but did not increase endocannabinoids above normal naïve levels. To further assess the in vivo relevance we analyzed the activity of the anti-inflammatory transcription factor PPARγ which is activated by cannabinoids and found that R-flurbiprofen increased PPARγ activity in DRGs and spinal cord (Fig. 4e). From these results, we infer that R-flurbiprofen caused a restoration of endocannabinoid levels, which were diminished following nerve injury, and therefore likely led to a better utilization of available CB1 receptors, without causing excessive cannabinergic activation. To confirm the lack of central CB1-mediated effects after R-flurbiprofen, we measured body temperature. Activation of brain CB1 receptors causes a characteristic hypothermia (temperature <35°C) (Fig. 4 f), a sensitive indicator of cannabinergic side effects. R-flurbiprofen had no effect on body temperature.

### Effects of R-flurbiprofen in SNS-CB1^−/−^ mice and effects of CB1 and CB2 receptor antagonists

To further evaluate whether the antinociceptive effects of R-flurbiprofen were mediated through normalization of endogenous cannabinoids in nociceptive neurons, we tested its antinociceptive efficacy in mice lacking the CB1 receptor selectively in primary afferent DRG neurons. These mice were generated as described [13] by mating mice carrying two CB1-floxed alleles (CB1^fl/fl^) [25] with mice that express cre-recombinase under control of the promoter of the SNS gene (SNScre) [13]. SNS codes for the voltage-dependent sodium channel Nav1.8, which is exclusively expressed in peripheral neurons of dorsal and trigeminal ganglia [26]. Following nerve injury, SNS-CB1^−/−^ mice show enhanced nociceptive behavior compared with that of CB1^fl/fl^-mice [13]. Treatment of both SNS-CB1^−/−^ and CB1^fl/fl^ with R-flurbiprofen resulted in a significant reduction of the nociceptive responses towards mechanical stimulation (*P* = 0.001 and *P*<0.001, respectively; Fig. 5 a, b). With respect to cold stimulation, R-flurbiprofen significantly reduced nociceptive behavior in CB1^fl/fl^ mice (*P* = 0.036), but not in SNS-CB1^−/−^ mice (*P* = 0.315). R-flurbiprofen restored anandamide levels in SNS-CB1^−/−^ and CB1^fl/fl^ mice to a comparable degree as in wild type animals (Fig. 5 c). To further test cannabinoid mediated effects we used specific antagonists at CB1 and CB2 receptors, AM251 and AM630, respectively (Fig. 5d, e, f). Mice were treated after SNI with R-flurbiprofen or vehicle. A combination of both drugs (3 mg/kg, single dose) was then injected i.p. in R-flurbiprofen treated mice (indicated by a black arrow). The cannabinoid receptor blockage completely abolished the antinociceptive effect of R-flurbiprofen in tests for heat (5d) and mechanical hyperalgesia (5e) and cold allodynia (5f), with partial recovery of R-flurbiprofen mediated antinociception within the following 2 days. The cannabinoid receptor antagonists did not evoke hypernociception at the respective doses in vehicle treated mice.

### R-flurbiprofen reduces microglia activation after nerve injury

**Figure 6:**
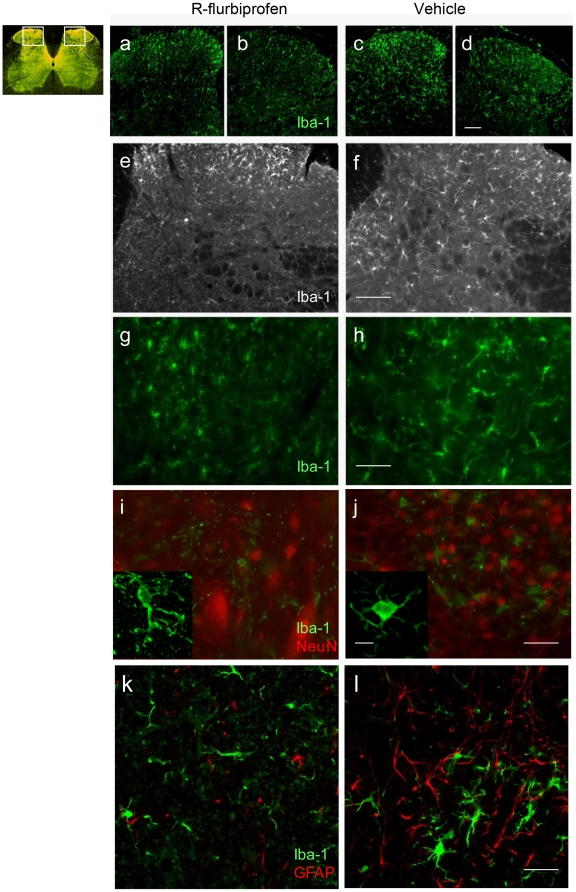
Immunofluorescence analysis of microglia in the spinal cord dorsal horn seven days after nerve injury. Microglia was identified by Iba-1 immunoreactivity (green). Mice were treated with R-flurbiprofen (4.5 mg/kg twice daily, left panel) or vehicle (right panel). Figures show the side ipsi lateral to the nerve lesion (**a, c, e, f, g, h, i, j, k, l**) and the contra lateral side (**b, d**). The inserts in i and j are z-projections. In I, J neurons were visualized by NeuN immunofluorescence (red). In k, l astrocytes were stained for glial fibrillary acidic protein (GFAP, red). Scale bars: 100 µm in a–f; 50 µm in g–l; 20 µm in the inserts of I, j.

**Figure 7:**
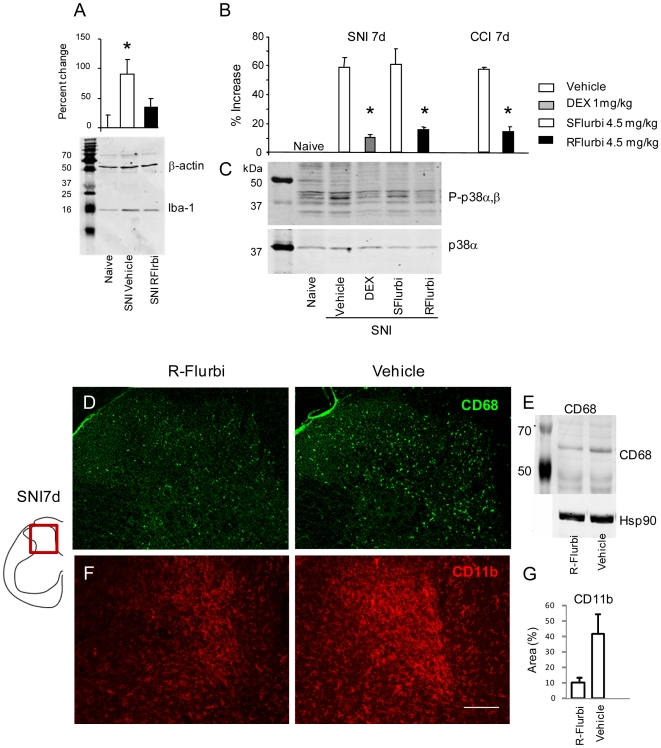
Quantitative analysis of microglia marker proteins in the dorsal horn of the spinal cord. **a** Western Blot analysis of Iba-1 immunoreactivity in the ipsi lateral L4/5 mouse spinal cord dorsal horn 7 days after SNI and treatment with R-flurbiprofen (4.5 mg/kg twice daily) or vehicle. Representative blot of n = 4 per group. B. The densitometric ratio of Iba-1 versus loading control β-actin was used for statistics; *P*<0.05. **b** Quantitative EIA analysis of phopho-p38 MAP kinase in the L4/5 mouse spinal cord dorsal horn ipsilateral to 7 days SNI or CCI and treatment with R-or S-flurbiprofen (4.5 mg/kg twice daily), dexamethasone (positive control) or vehicle. **c** Respective representative Western Blot of phospho-p38 and p38 alpha. **d** Immunofluorescence and **e** Western Blot analysis of microglia CD68 immunoreactivity in the spinal cord dorsal horn 7 days after nerve injury and treatment with R-flurbiprofen (4.5 mg/kg twice daily) or vehicle ipsi lateral to the nerve lesion (n = 3 per group). **f, g** CD11b immunofluorescence of dorsal horn microglia and densitometric CD11b image analysis in the ipsilateral dorsal horn 21d after SNI. Scale bar: 100 µm.

As described above, R-flurbiprofen normalized endocannabinoid levels in the DRGs, most likely leading to improved neuronal CB1-mediated antinociceptive effects. As the deficiency of the CB1 receptors in peripheral nociceptive neurons of the DRG did not completely abolish the antinociceptive effect of R-flurbiprofen, whereas a combination of a CB1 and a CB2 inhibitor accomplished full antagonisms, we further investigated potential effects at CB2 receptors. CB2 is expressed on microglia in the spinal cord [27]. The activation of spinal cord microglia after peripheral nerve injury contributes to the development of neuropathic pain [28]. Agonists at CB2 receptors presumably prevent this activation by inhibition of cytokine production [29]. We therefore analyzed effects of R-flurbiprofen in terms of microglia activation. We found the typical microglia activation after nerve injury, as assessed by immunoreactivity for Iba-1 (Fig. 6, Suppl. Fig. S1 c), CD68 (Fig. 7 d, e) and CD11b (Fig. 7 f, g) in vehicle treated mice and mitigated in R-flurbiprofen-treated mice. Overall Iba-1 immunoreactivity was slightly reduced with R-flurbiprofen treatment (Fig. 7 a) as revealed by Western Blot analysis on spinal dorsal horn tissue. More strikingly however, R-flurbiprofen-treated mice predominantly demonstrated microglia with a ramified morphology whereas the control animals presented with the typical amoeboid microglia, indicative of the activated phagocytic phenotype (Fig. 6 i, j and inserts, k, l). In addition, R-flurbiprofen significantly reduced the activation of p38 MAP kinase in the spinal cord dorsal horn as revealed by quantitative phospho-p38 analysis (Fig. 7 b, c). Phospho-p38 is considered to be a marker for activated microglia [30], [31], [32], [33], [34], [35], [36]. S-flurbiprofen had no effect on phopho-p38, supporting the specific role of the R-enantiomer. R-flurbiprofen strongly reduced the nerve injury evoked increase of CD68 and CD11b immunoreactive microglia in the dorsal horn (Fig. 7d–g). CD11b is part of the complement 3 receptor and is a marker for microglia. CD68 is a transmembrane 37 kDa glycosylated protein expressed by tissue macrophages and activated microglia [36]. It plays a role in phagocytic activities of tissue macrophages, both in lysosomal metabolism and extracellular cell-cell interactions. Depending on glycosylation molecular masses differ. In brain and spinal cord extracts the antibody detects a band at about 65 kDa in Western Blot analyses according to the data provided by the manufacturer.

R-Flurbiprofen also reduced the nerve injury-evoked activation of astrocytes in the dorsal horn (Suppl. Fig. S2). Astrocytes express CB1 receptors and are activated by endocannabinoids released by neurons [37]. Astrocytic CB1 activation in vitro modulates phospholipase C, MAP kinase pathways and chemokine release from astrocytes [37], [38], which contribute to the enhancement of neuropathic pain [39], [40].

## Discussion

We demonstrate that R-flurbiprofen reduces nociceptive behavior in two models of neuropathic pain by restoring diminished endocannabinoid levels in nociceptive neurons and pain associated brain centers. The nerve injury-evoked endocannabinoid reduction resulted from an imbalance of endocannabinoid synthesis and breakdown after axonal injury. Our data suggest that by restoring endocannabinoids, R-flurbiprofen allows for a better utilization of endogenous pain defense mechanisms mediated through the neuronal CB1 as well as the microglial CB2 receptors.

Because of the combined increase of peripheral CB1 receptor activation and CB2-mediated silencing of activated microglia, R-flurbiprofen retained its antinociceptive efficacy despite a temporal downregulation of CB1 receptors in primary afferent neurons after axonal injury. As R-flurbiprofen is not a direct agonist at CB1 or CB2 receptors but appears to act by increasing endocannabinoid levels, its effects are unlikely to be subject to the development of centrally mediated psychotropic side effects, supported by the lack of changes in body temperature which is the most sensitive parameter in rodents for typical CB1-mediated effects in the brain. Increased synthesis of NAPE, the precursor of anandamide was suggested to represent a cytoprotective response in relation to various forms of neurotoxicity [41]. We therefore propose that R-flurbiprofen may be a promising analgesic treatment in patients with neuropathic pain or an interesting lead compound in the research of endocannabinoid-modulating drugs.

Our results revealed a disturbance of the endocannabinoid mediated pain defense following axonal injury, which may result from the loss of the axonal transport of growth factors from the innervated target organ to the neuronal body. A similar disturbance has been demonstrated previously for the endogenous opioid system [42]. The additive effects of disturbances of both the opioid and endocannabinoid-mediated endogenous inhibition of pain probably contribute to the therapeutic difficulties in achieving sufficient pain relief, particularly for intractable clinical manifestations such as cold and tactile allodynia. The observed deregulation would be also compatible with the astonishing benefits frequently provided by cannabinoid treatment in patients with immune cell and microglia activation and auto-immune mediated neuronal pathology, such as in multiple sclerosis [43], [44] or Huntington's disease [45]. Interestingly, a similar decrease of anandamide as a result of increased FAAH activity was recently found in the paw skin in bone cancer bearing mice [12]. However, in contrast to nerve injury, bone cancer was associated with an increase of CB1 receptor expression in the DRGs [12] as previously shown for inflammatory models [13], suggesting that normalization of AEA disturbances through FAAH inhibition may be even more effective in the relief of cancer pain than neuropathic pain.

We found no effect of S-flurbiprofen on nociceptive behavior or microglial activation as assessed by phopho-p38 activity. In addition, S-flurbiprofen exerted considerable gastrointestinal toxicity due to potent cyclooxygenase inhibition. We can exclude that the effects of R-flurbiprofen require cyclooxygenase inhibition. The observed effects are also unlikely to depend on the previously shown NF-κB inhibition [5], which rather exerts neuroprotection in dorsal horn neurons after peripheral nerve injury [46], than promotion of neuroinflammation [47]. The effects of R-flurbiprofen on the microglia phenotype and activation state were stronger than suggested by the exclusive modulation of anandamide in the dorsal horn. However, the subsequent activation of PPARγ may further fortify the anti-inflammatory effects.

Since R-flurbiprofen also modulates gamma-secretase dependent substrate processing [48] it was considered as potential drug for slowing the progression of Alzheimer's disease [7], [49]. It is unknown if this is mediated through modulation of endocannabinoids and if gamma-secretase is somehow involved in the neuronal adaptations and glia activation that occur after peripheral nerve injury.

In summary we demonstrated that R-flurbiprofen restored endogenous cannabinoids that are diminished in DRGs and spinal cord dorsal horn after a peripheral nerve injury. R-flurbiprofen thereby enhanced the endogenous defense against neuropathic pain, without direct cannabinoid receptor stimulation, therefore presumably without tolerance or CB1-mediated central side effects. The apparent lack of a specific target may well be an advantage because it helps to avoid significant toxicity. R-flurbiprofen is an interesting drug for patients with neuropathic pain and may represent a lead compound for further research in endocannabinoid-modulating-drugs.

## Supporting Information

Figure S1 Time course of mRNA levels of cannabinoid-1 receptor (CB1) in the (a) L5 DRGs and (b) the dorsal horn of the lumbar spinal cord ipsi lateral to a sciatic nerve lesion in three different models of neuropathic pain, i.e. the spared nerve injury, SNI, the chronic constriction injury, CCI and the spinal nerve ligation, SNL analyzed by Affymetrix U34 microarray in triplicate. Pooled samples of three animals each were used. The asterisks indicate statistically significant results with P<0.05. 1c Composite image of the spinal cord 7 days after nerve injury in animals treated with vehicle. CB1 receptor red, microglia Iba-1 green, neuronal NeuN blue.
(0.11 MB PDF)

Figure S2 Immunofluorescence of the glial fibrillary acidic protein (GFAP) as a marker for astrocytes in the dorsal and ventral horns of the spinal cord ipsilateral to a peripheral sciatic nerve injury. Rats were dissected 4 weeks after the nerve injury (SNI model) and 14 µm sections of the L4/5 spinal cord were cut on a cryotome, incubated with anti-GFAP antibody and Alexa488-labeled secondary antibody. Images were captured on a Nikon fluorescent microscope. R-Flurbiprofen was administered twice daily by i.p. injection (4.5 mg). Treatment was initiated one day after nerve injury.
(0.10 MB PDF)
