# Supplementary figures and images for: R-Flurbiprofen Reduces Neuropathic Pain in Rodents by Restoring Endogenous Cannabinoids
Source: PLoS One. 2010 May 13;5(5):e10628. doi: 10.1371/journal.pone.0010628 (PMC2869361; doi:10.1371/journal.pone.0010628)

Suppl. Figure S1

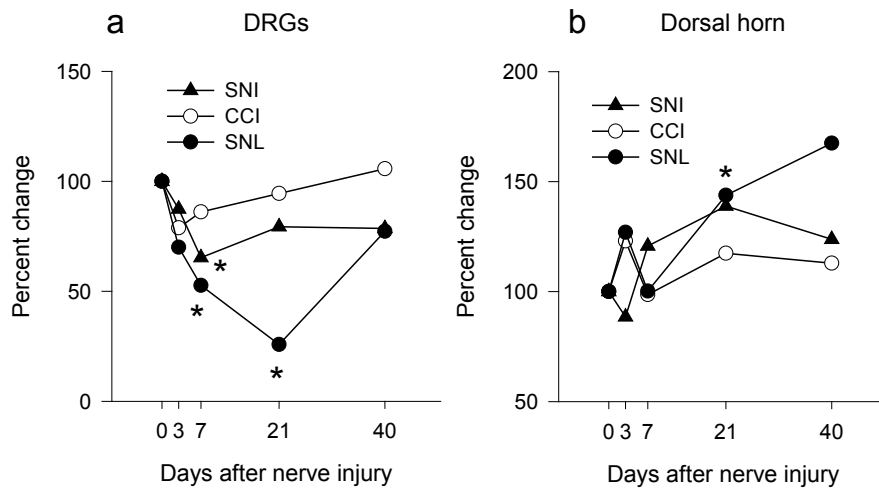

Suppl. Figure S1c

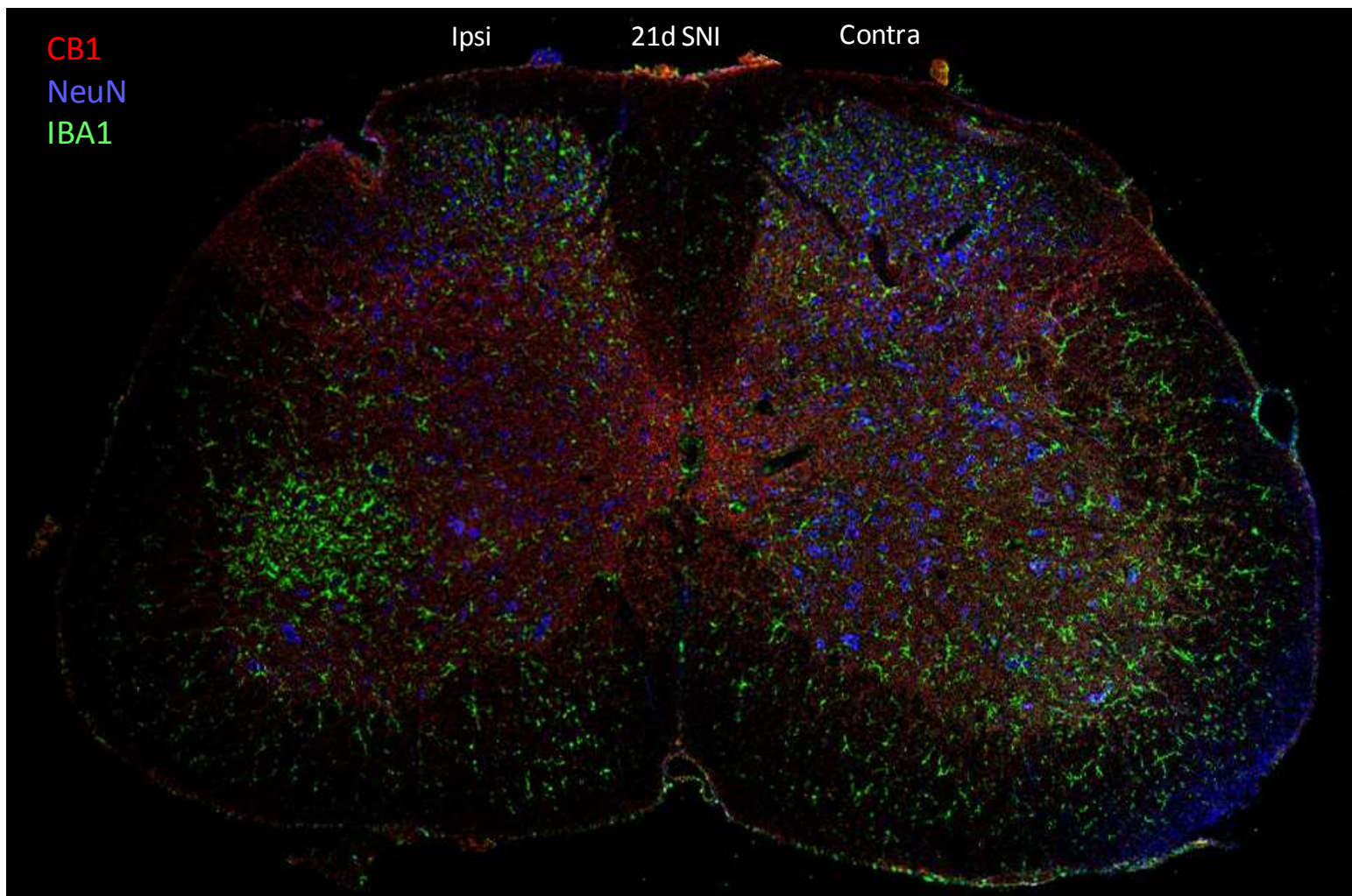

Supplement: Figure S1 — Time course of mRNA levels of cannabinoid-1 receptor (CB1) in the (a) L5 DRGs and (b) the dorsal horn of the lumbar spinal cord ipsi lateral to a sciatic nerve lesion in three different models of neuropathic pain, i.e. the spared nerve injury, SNI, the chronic constriction injury, CCI and the spinal nerve ligation, SNL analyzed by Affymetrix U34 microarray in triplicate. Pooled samples of three animals each were used. The asterisks indicate statistically significant results with P<0.05. 1c Composite image of the spinal cord 7 days after nerve injury in animals treated with vehicle. CB1 receptor red, microglia Iba-1 green, neuronal NeuN blue. (0.11 MB PDF) [file pone.0010628.s001.pdf]

Suppl. Figure S2

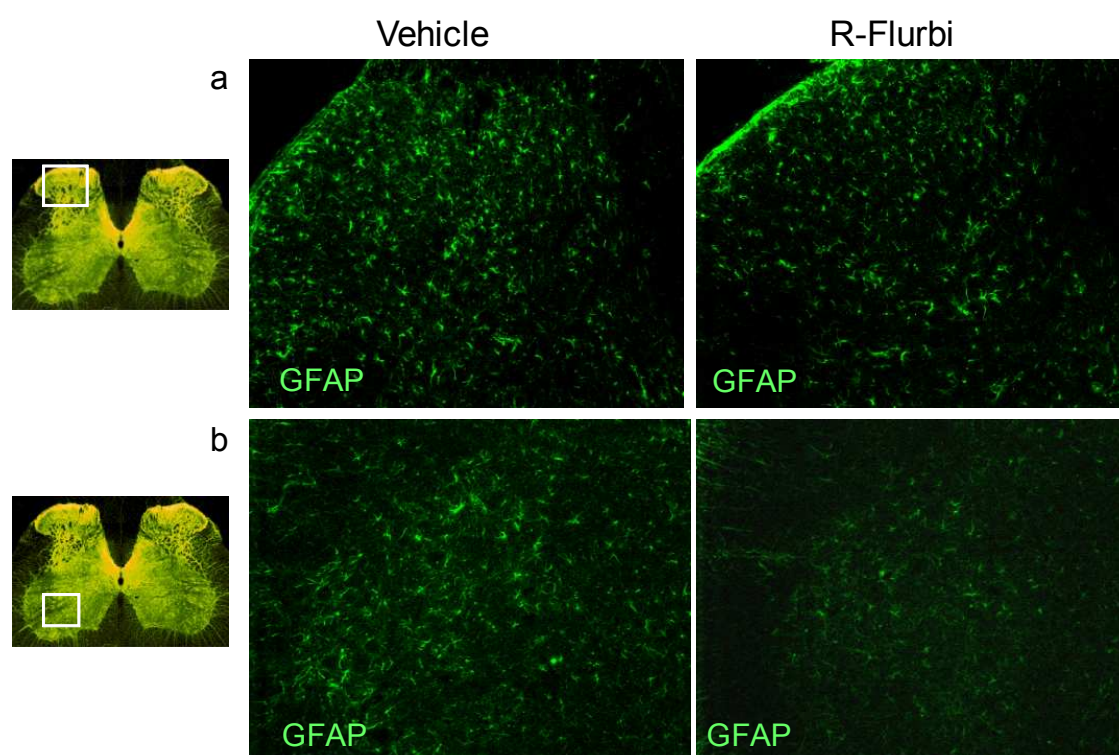

Supplement: Figure S2 — Immunofluorescence of the glial fibrillary acidic protein (GFAP) as a marker for astrocytes in the dorsal and ventral horns of the spinal cord ipsilateral to a peripheral sciatic nerve injury. Rats were dissected 4 weeks after the nerve injury (SNI model) and 14 µm sections of the L4/5 spinal cord were cut on a cryotome, incubated with anti-GFAP antibody and Alexa488-labeled secondary antibody. Images were captured on a Nikon fluorescent microscope. R-Flurbiprofen was administered twice daily by i.p. injection (4.5 mg). Treatment was initiated one day after nerve injury. (0.10 MB PDF) [file pone.0010628.s002.pdf]
